# The untapped potential for healthcare to support recovery for patients with stress-related exhaustion disorder – creating an experience of generalised safety

**DOI:** 10.1080/02813432.2025.2480869

**Published:** 2025-04-01

**Authors:** Susanne Ellbin, Agneta Lindegård, Fredrik Bååthe

**Affiliations:** a Institute of Stress Medicine at Region Västra Götaland, Gothenburg, Sweden; b School of Public Health and Community Medicine, Institute of Medicine, Sahlgrenska Academy at University of Gothenburg, Gothenburg, Sweden; c Institute for Studies of the Medical Profession, LEFO, Oslo, Norway

**Keywords:** recovery from stress-related exhaustion disorder, generalised unsafety theory of stress, patients experience of healthcare encounter, primary care, qualitative study

## Abstract

**Background:**

Exhaustion disorder (ED) is one of the most rapidly increasing causes of sick leave in Sweden. The prolonged recovery time from ED creates a dilemma on both the societal and individual level. How patients experience the encounter with health care is critical for the recovery from ED. The aim of this study was to explore how patients with ED experience the encounter with health care.

**Method:**

Data from 23 semi‑structured interviews with ED patients were analysed using content analysis.

**Results:**

One of the main findings was that patients want to be listened to and taken seriously in the encounter with health care. However, patients experience that healthcare struggles to meet these expectations. Several informants reported not being listened to, worrying symptoms were overlooked, an individualised care plan was lacking, and patients experienced that they were prematurely dismissed. This created an experience of unsafety, and that could inadvertently maintain the stress response and negatively influence recovery for patients with stress‑related disorders.

**Conclusion:**

It is paramount to convey an experience of predictability, despite the fact that patients undergo an unpredictable process related to their ED illness. By combining the traditional load‑recovery theory with the generalised unsafety theory of stress, we can tap into the potential to enhance recovery for patients with ED. We suggest that if the encounter with healthcare provides an experience of generalised safety, the conditions for patients with stress‑related disorders to recover would be distinctly enhanced.

## Background

The increase in sick leave due to workplace stress is a general trend seen across Europe, and the societal costs are high [1]. In Sweden, stress-related diagnoses have been the fastest growing cause of sick leave since 2010, according to the Swedish Social Insurance Agency [2]. The diagnosis “exhaustion disorder” (ED) has been established as a formal medical diagnosis in Sweden since 2005 under the code F 43 8 A in the ICD-10 coding system [3]. This disorder is characterised by a variety of symptoms, including extreme physical and mental fatigue, sleep disturbance, cognitive impairment, and reduced stress tolerance, often in combination with symptoms of depression, anxiety, and somatic symptoms such as non-generalised musculoskeletal pain, chest pain, and gastrointestinal problems [4,5]. Patients with ED share many cardinal symptoms with individuals who meet the criteria for burnout, which is included in the 11th revision of the International Classification of Diseases (ICD-11) and is described as ‘a syndrome resulting from chronic workplace stress that has not been successfully managed’ [6]. This differs from the ED diagnosis, which can be related to stress exposure both in the workplace and in private life [3,7].

In Sweden, primary healthcare serves as the first line of contact for patients with non-emergency health concerns, including mental health problems. Primary care encompasses a wide range of healthcare services, including various units. Patients with ED primarily seek care and are treated at two of these units: primary care centers—mainly staffed with physicians, registered nurses and psychologists—and rehabilitation centers, staffed with physiotherapists and occupational therapists. Patients diagnosed with ED present a complex array of physical, cognitive, and emotional symptoms [8]. Clinical treatment recommendations for ED include a variety of interventions, such as balancing activation and recovery, relaxation techniques, psychotherapy, and specific return-to-work interventions [9].

Despite the fact that ED has been a formal diagnosis in Sweden since 2005, there is still a limited body of evidence about what constitutes the most effective treatment strategy [10,11]. Previous research has produced limited evidence and raised concerns about inconsistencies in the effectiveness of treatments, which can create a sense of uncertainty among healthcare professionals [12,13]. Uncertainty among caregivers has been found to negatively impact trust among patients in their encounters with healthcare professionals [14–16]. In fact, previous studies found that creating a trustful encounter is essential for treatment outcomes and satisfaction with care [17–19].

The importance of patient trust in the healthcare encounter can be further understood by applying the generalized unsafety theory of stress [20]. Brosschot et al. suggest that the human stress response is constantly engaged, until there are clear indications of safety. Their theory provides a way to understand how patient’s experience of trust and safety in the encounter with healthcare is critical to prevent prolonged recovery from stress-related disorders. In line with this research, ED patients’ experiences in the encounter with healthcare is essential for their recovery process. Hence, the aim of this study was to explore how patients with an ED diagnosis experienced the encounter with primary healthcare.

## Materials and methods

### Methods

The study has a qualitative design, relying on interviews to capture multifaceted nuances from ED patients’ encounters with healthcare [21].

### Informants and setting

The informants were recruited through advertising on social media, specifically Swedish Facebook groups for patients with ED, and through the website of the Institute of Stress Medicine (the R&D unit responsible for the study). Inclusion criteria were that the participant currently had or previously had a diagnosis of ED and did not have other psychiatric illnesses such as bipolar disorder or alcohol dependence. Comorbidity regarding depression and anxiety was allowed. Another inclusion criterion was that the informant was able to understand and speak Swedish. To ensure the inclusion and exclusion criteria, a screening interview was designed and conducted by the first author (SE) and the second author (ALA). In connection with this, an appointment was scheduled for an interview. The study included 23 individuals, 18 women and 5 men. The age ranged between 32 and 61, with an average of 51 years. The average years of education was 16 years (range 11–20 years), and professions reported were teacher, economist, nurse, assistant nurse, systems scientist, lawyer, building antiquarian, optician, salesperson, librarian, manager, architect, and pharmacist.

Ten of the informants were still in treatment for ED, and 13 had completed treatment. The time since the last episode of ED was between 0 to 11 years, with an average of 5 years. Sixteen of the informants had experienced one or more relapses due to ED, resulting in a new period of sick leave. The informants mainly received their initial care at two units within primary care, primary care centres, and rehabilitation centres. Most informants had recovered to the point that they were able to return to work. For more information about the informants, see Table 1.

**Table 1.** Information about the informants.

Informant	Female/male	Age	Education years
1	f	61	16
2	f	38	17
3	f	43	18
4	f	48	17
5	f	55	20
6	f	44	16
7	m	58	16
8	f	48	16
9	f	59	17
10	f	45	12
11	f	54	14
12	m	35	–
13	m	32	20
14	f	50	12
15	m	60	13
16	f	61	14
17	f	61	11
18	m	58	14
19	f	52	17
20	f	60	20
21	f	56	17
22	f	55	17
23	f	46	16

## Data collection

A semi-structured interview guide was used for the interviews. The guide explored experiences from the encounter with healthcare when seeking care for stress-related symptoms. A translated interview guide is available in Appendix A. The informants could choose to be interviewed either face to face (4 informants) or *via* Zoom (19 informants). All interviews were conducted by the first author, a psychologist with over 40 years of clinical experience and 12 years of experience treating patients with ED.

The interviews were performed during spring/summer 2022. Each interview was scheduled for 60 min and was audio recorded and transcribed verbatim by an experienced researcher who was not otherwise involved in the study. Notes were also taken by the interviewer during the interview. A total of 222 pages of transcribed text were available for the analysis. Before the interview, the informants were informed that the purpose of the interview was to explore their experiences of the encounter with healthcare when seeking care for stress-related symptoms. They were also informed about confidentiality, and all informants signed an informed consent before entering the study.

## Data analysis

Since there are few previous qualitative studies exploring different dimensions of the encounter with healthcare when seeking care for stress-related symptoms, inductive content analysis was chosen according to Elo and Kyngäs [22]. The first author (SE), who conducted all interviews, worked with the initial analysis by first becoming acquainted with the material and getting an overview by reading all interview transcripts. In the subsequent analysis, authors number 2 (ALA) and number 3 (FB) read through the verbatim transcripts. Each author wrote reflections and summary notes and systematically coded the interview. The individual codes were shared and combined during multiple conversations between the group of authors, and initial categories were subsequently formed. The analytical process continued by further refining and defining the categories in a recursive process to identify underlying patterns of shared meaning that enabled the authors to combine several of the initial categories into richer and more complex categories. When naming the final categories, we aimed to create an inclusive description to convey the multifaceted nuances.

All categories are exemplified with rich quotations from the interviews and are presented under two categories. This provides a time sequence, as seen from the informant’s view, in relation to the encounters with healthcare.

The research group consisted of three persons. The first author (SE) is a psychologist with over 40 years of clinical experience and 12 years of experience treating patients with ED. The second author (ALA) is a senior researcher and physiotherapist with 40 years of experience in the profession. The third author (FB), also a senior researcher, with a PhD in medicine and an original education in industrial engineering and management, with many years in managerial roles, including head of department at a Hospital in Sweden. So as a research group we cover many dimensions, and as such the analytical process has been both multi-professional and trans-disciplinary.

## Ethical considerations

The study was conducted in compliance with the Declaration of Helsinki and was approved by the Swedish Ethical Review Authority (Dnr 2021-06505-01). All informants signed informed written consent before the interview.

## Results

The analysis resulted in two main categories: The Healthcare Encounter and the Care Process. Each category is illustrated by quotations and described below along with its associated subcategories. The categories are organised in a temporal continuity, conveying how the informants experienced the encounter with healthcare when seeking care for stress-related symptoms.

## The healthcare encounter

### Expectations of being acknowledged and taken seriously

Each informant had encountered several healthcare professionals within the primary care context. Some of the informants described being treated by healthcare professionals who have previous experience with ED patients. In this context, physicians were mentioned who were empathetic, acknowledged patients symptoms and conveyed an experience of being taken seriously by providing clear guidelines and recommendations for rehabilitation. There were also mentions of physicians who supported with well-written sick leave certificates, facilitating the bureaucratic process with the Social Insurance Agency,

I had a wonderfully understanding and knowledgeable general practitioner. Someone who had the knowledge and experience, understood what this was about, and in some way took responsibility and lifted, how should I put it, the burden from me. He made it clear that there was no alternative. ‘You have to be on sick leave! And now you let go of everything, and we’ll see each other again in, I don’t remember exactly, maybe 3 weeks or so to start with. [8]

Many of the informants reported that their condition was difficult to describe and understand, both in terms of the symptoms and origin. Additionally, the informants often sought healthcare for various physical symptoms before receiving an ED diagnosis. They rarely had any specific or detailed expectations about the healthcare encounter. However, there was a clear expectation that they would be acknowledged and taken seriously. Several informants expressed a sense of helplessness. Their needs included being cared for and talking to someone who understands them, who can help make sense of their different symptoms and can guide them toward recovery.

Take care of me! I don’t know anything; I can’t do anything. So that feeling. I almost couldn’t connect to how I felt. [9]

To have someone who listens attentively and is caring. Someone who maybe, yes, someone who perhaps gives you a little hug and says, ‘I understand.’ Something like that. [11]

### Experiences of not being taken seriously

However, most informants conveyed experiences of being diminished or not being taken seriously. When symptoms were neglected or downplayed by a healthcare professional, some of the informants described that this led to the aggravation of their symptoms, since the experience of being disregarded made them believe they were not ‘sick enough’ and therefore tried to carry on with their daily lives despite their symptoms.

The experience of not being taken seriously was further emphasised in the informants’ reports that healthcare professionals did not always have sufficient knowledge to provide adequate advice and support. As a result, they sometimes did not seek care again even when symptoms worsened, or they decided to seek care in another primary care centre or elsewhere.

I had physical symptoms for many years on and off, but the healthcare [staff] made me ignore everything because I was always told: Cause unknown, could be stress. [8]

And then she [the physician] said: ‘Yes, no, but I can’t find anything’ …  … but I see that you have come here for fatigue before and there are always patients who look for causes. And we can’t burden the healthcare [system] with something like this. [11]

As the physician said: ‘You are not healthy, but we can do no more for you, so now we are sending you home’. I felt like I had been dumped in a dustbin. [20]

What I can say is if you look at the primary care centre, it seems like they have very little knowledge and little time for exhausted patients in particular. They seem not to have the time to ask me: ‘What has actually happened that brings you here today … .’ [19]

## The care process

### What was considered valuable regarding treatment interventions varied from person to person

The treatments offered to the informants varied substantially, as did the actual care process. Many of our informants initially sought care at their selected primary care centre. However, only a few informants (4 out of 23) reported that they had received all treatments there. Many of the informants had searched for other alternatives on their own. The most common approach was to seek private alternatives, for example, a private psychologist or multimodal treatment in special treatment units.

What was considered valuable regarding treatment interventions varied from person to person. Different informants highlighted different components that had been most valuable to them. A common remark from the informants was that treatment interventions should not be too standardised, and they expressed a desire for healthcare professionals to acknowledge their unique needs and provide individualised care.

There were also certain dimensions of the treatment that were highlighted by the informants. They reported that having someone supportive to talk to and finding a method for relaxation were important treatment components. Learning mindfulness, yoga, or basic body awareness provided a method to ‘calm the system’ and get to know one’s body, and this was seen as an important part of the rehabilitation process. Just as often, informants highlighted the importance of psychotherapy, with the aim of understanding the underlying problem and establishing new strategies. Several other interventions were mentioned by informants as valuable, including group-based interventions and interaction with others. Many informants described that they benefited from the different treatments, but they rarely reported that one specific treatment contributed to the actual recovery process, thus enabling them to return to work.

Yes, the turning point has been the physical therapy … and psychologist … for my part it was about my behaviour … start by saying no … .and then magnesium … exercise. [9]

Changed diet … supplements, focus on sleep and recover. [12]

Medi yoga. Because I see things metaphorically as I do, my actions, my behaviours and how I move my body. [13]

Finding underlying problems really. I hadn’t understood that the problems were so connected. [19]

### Lack of psychosocial perspectives

Several informants stated that the healthcare system was primarily focused on the bio-medical perspective of their disease and expressed that they lacked a more comprehensive perspective where their whole life situation was included. Moreover, they expressed that healthcare professionals need to have a greater focus on all dimensions of the ED diagnosis, both in relation to private life and working life. Some expressed a desire to receive questions about their whole life situation, as well as practical advice on how to cope with daily life and routines such as sleep, food, exercise, and personal relations. Furthermore, the informants expressed that it would have been helpful if healthcare professionals had ‘asked the right questions’ to create a reasonable understanding of the important underlying factors for the onset of their ED disorder.

The more comprehensive approach has not been there …  yes, let’s try mindfulness, but there is so much more, like a more holistic approach: What does your life look like? Do you exercise? So, in a way, I mean the totality of the family situation. [4]

What I wish is for healthcare [professionals] to look at how you handle the social [situation] at home, how you feel, how you live. [9]

### Lacking a care plan and being prematurely dismissed by healthcare professionals

A recurring theme in the interviews is that the informants wanted more help from healthcare professionals so that they could better understand their illness, and the informants felt that there was a lack of knowledge about ED among healthcare professionals. Many informants felt confused about what was needed to recover, and some expressed that the healthcare staff also seemed to show confusion and uncertainty. Several informants also expressed a need for healthcare professionals to provide a structured plan they could follow during the care process, pointing out that continuity and follow up were missing. Many of the informants stated that the primary healthcare centre eventually exhausted all treatment options, and this often occurred long before the informants felt that they had sufficiently recovered. This resulted in a sense of having been dismissed from healthcare. The informants emphasised that the healthcare system ultimately transferred responsibility for continued care to the patients themselves after primary care exhausted all of its options. This means that continued care and coordination will depend on the patient’s ability to take the initiative and navigate the complex healthcare system.

Yes, there must be a plan, even though I received help with counselling, then you were sort of thrown out, yes, then it was over with. [11]

I think there is a lack of an action plan. They don’t know, after they prescribe antidepressants, after you have received CBT (cognitive behavioural therapy). Then they don’t do much. [12]

It shouldn’t be that difficult to be able to have a clearer structure around it, or that there is something like an action plan … . [6]

I had had to see four junior physicians, I think, and had to sort of repeat my background and history. This sort of constantly getting in touch with new physicians, it felt a bit like I wasn’t that important. That was the feeling you got, that this was not serious enough. [2]

The healthcare [system] has strategies and guidelines, and they are very similar, but the reason why you are exhausted can be so incredibly different. [2]

### The return-to-work process

For many of the informants, the return to work was a process where they felt pressured, knowing that it was the final step in the care process. The informants were aware that the health insurance regulations pressured physicians to quickly get patients back to work, although the conditions at the workplace often contributed to the problem. This experience adversely impacted trust in the patient-physician relationship. Several informants talked about how the pressure to return to work too early, in addition to the feeling of vulnerability, led to relapses and delayed their overall recovery.

It can feel like I’m just someone who needs to recover because I have to return to work. [22]

I would have appreciated it if I didn’t feel like they were just trying to rush me back to work. Instead, I wished they had a proper treatment plan in place. [3]

I understand that you shouldn’t provide very long sick leave for people, but it was a little difficult to be taken seriously …  Yes, but a month is probably enough for you, and then you are to go back to work. And that did not work for me. [2]

## Discussion

One of the main findings of this study, as expressed by the informants, was the need to be listened to and taken seriously in the encounter with healthcare professionals while dealing with their multifaceted stress-related condition. However, our result showed that, in many cases, primary healthcare centres fail to meet these expectations. Instead, informants experienced how their symptoms, worries and concerns were not listened to and taken seriously in the encounter with health care. Moreover, the experience of being prematurely dismissed from healthcare contributed to a never-ending search for care that would lead to recovery. In addition, several informants reported that worrying symptoms were dismissed and that they lacked an individualised care plan.

These results are troubling, since the ability of healthcare professionals to listen to patients and show an interest in their wellbeing has been shown to be crucial for how patients perceive the quality of the care they receive [23,24], and this can have an effect on both treatment results and clinical management [17].

### Towards a more sustainable recovery – addressing informants experiences of unsafety

The foundational hypothesis driving clinical practice is the load-unload-recovery theory [25]. Hence, as a clinician, you help the patient to identify and unload the most severe stressor, with the explicit intent of facilitating the patient’s recovery. Sick leave, along with the interventions offered by healthcare plays a significant role in the traditional ED recovery process. However, the results in this study suggest that this is not enough. The ability of healthcare professionals to create a sense of safety in the encounter with the patient is an important piece of the puzzle that can further help ED patients recover from their illness.

The generalised unsafety theory of stress introduced by Brosschot et al. [20], suggests that the stress response is a default state. Disengaging this stress response only happens when there are clear signals that safety is being provided. According to the results of the present study, patients experienced a feeling of unsafety in the encounter with healthcare. In line with the theory presented by Brosschot et al. this experience of unsafety could inadvertently maintain the stress response and negatively influence treatments and rehabilitation for patients with stress-related disorders. The importance of creating an experience of safety in the healthcare encounter can further be understood by considering Almén, who suggests that the ED diagnosis entails a range of particular concerns creating unsafety, such as what does the diagnosis means, how long will the illness last, and what does recovery mean? [26]. Thus, we argue that it is not sufficient for healthcare to address and eliminate stressors when treating patients with ED, it is equally important to provide an experience of safety in order to create enhanced conditions for recovery. This implies that one of the central goals in the encounter with patients should be to convey an experience of safety. Yet, the theory by Brosschots et al. has not received enough recognition to be widely integrated in clinical practice.

We suggest that if healthcare would be able to provide and convey an experience of generalised safety, the conditions for recovery from ED would be improved among patients. Thus, it is about conveying the message that ‘healthcare professionals are here for you, listening and taking you seriously, and healthcare is providing a structured written plan with explicit follow up dates to reduce uncertainty and enhance predictability’. The combinational effect – where we continue to use traditional load-recovery theory combined with the more recent generalised unsafety theory of stress and adjust practice accordingly – addresses the societal and individual need to find evidence-based approaches that enable a more sustainable recovery process for patients with stress-related disorders.

Taken together, the findings in this study, which explores patients’ experiences in the encounter with healthcare, can be considered hopeful as they point to an untapped potential to enhance recovery. By ensuring that the encounter between healthcare and the ED patient provides enough clear signals about safety, the individual stress system for each patient is likely to become deactivated, and recovery is facilitated further.

## Methodological considerations, strength and limitations

We used a structured interview guide to manage pre-understanding, minimize the risk of bias, and ensure that the informants’ experiences were accurately represented. To enhance the rigor of the analysis, reflections and discussions were conducted collaboratively, involving the multiprofessional group of authors. The interpretations of the data were kept as close as possible to the original empirical material.

Although a relatively large number of interviews were conducted, the sample was recruited through advertising on social media, specifically Swedish Facebook groups for patients with ED. This introduces a potential bias regarding which patients chose to participate. For instance, patients with negative care experiences might have been more inclined to participate to voice their dissatisfaction. We are also aware that the fact that the interviewer is a psychologist may have influenced the informants to highlight the psychological counseling as particularly helpful. Additionally, gender distribution was not specifically accounted for during recruitment, resulting in an overrepresentation of women in the study. At the same time this reflects the typical gender distribution for the ED diagnosis.

## Conclusions and clinical implications

We conclude there is an untapped potential to alter the troubling situation of long-term sick-leave among patients diagnosed with ED. By combining the traditional load-recovery theory with the generalised unsafety theory of stress, we can tap into the potential to enhance recovery for patients with ED.

We suggest that if the encounter with healthcare provides an experience of generalised safety, the conditions for patients with stress-related disorders to recover would be distinctly enhanced. Perceived safety is more than the perceived absence of threat. It is paramount to convey predictability, despite the fact that patients undergo an unpredictable process related to their ED illness. This means that healthcare needs to ensure the encounter with the patient communicates the following three things, at a minimum:

1. Healthcare is here – listening to your concerns and taking you seriously.
2. Healthcare is here – providing a framework for what to expect with an ED diagnosis, including personalised care with active follow-ups.
3. Healthcare is here – providing explicit information about the time and place for the next meeting.

## Appendix A. Interview guide

1. Describe how you experienced the encounter and treatment you received from the healthcare system for your exhaustion disorder?
2. Describe what was valuable to you and how you benefited from it in your recovery?
3. Which symptoms have you received help with, and which have you not received help with?
4. Describe whether you received treatment at the right time in your illness.
5. What expectations did you have regarding what the healthcare system could help you with?
6. What do you base your expectations on? (Something from others in similar situations, media, social media)
7. Is there anything in your treatment that you found less significant or unnecessary? In what way?
8. Is there anything you missed? How would you have been helped by what you missed?
